# The Anatomy of the Atrioventricular Node

**DOI:** 10.3390/jcdd12070245

**Published:** 2025-06-26

**Authors:** Robert H. Anderson, Damián Sánchez-Quintana, Jorge Nevado-Medina, Diane E. Spicer, Justin T. Tretter, Wouter H. Lamers, Zihan Hu, Andrew C. Cook, Eduardo Back Sternick, Demosthenes G. Katritsis

**Affiliations:** 1 Biosciences Division, Newcastle University, Newcastle-upon-Tyne NE2 4HH, UK; 2 Department of Human Anatomy and Cell Biology, Faculty of Medicine, University of Extremadura, 06006 Badajoz, Spain; sanchezquintana55@gmail.com (D.S.-Q.); jnevadomedina@gmail.com (J.N.-M.); 3 Heart Institute, Johns Hopkins All Children’s Hospital, St. Petersburg, FL 33701, USA; spicerpath@hotmail.com; 4 Division of Pediatric Cardiology, Cleveland Clinic Children’s, and The Heart, Vascular, and Thoracic Institute, Cleveland Clinic, Cleveland, OH 44195, USA; trettej3@ccf.org; 5 Department of Anatomy & Embryology, Maastricht University, 6211 LK Maastricht, The Netherlands; wouterhlamers@gmail.com (W.H.L.); zihan.hu@maastrichtuniversity.nl (Z.H.); 6 Institute of Cardiovascular Science, University College, London WC1E 6BT, UK; a.cook@ucl.ac.uk; 7 Electrophysiology Unit, Biocor Instituto, Nova Lima 34006083, Brazil; eduardosternick@gmail.com; 8 Department of Cardiology, Hygeia Hospital, 15123 Athens, Greece; dkatrits@dgkatritsis.gr; 9 Johns Hopkins Hospital, Baltimore, MD 21287, USA

**Keywords:** compact node, inferior extensions, fast pathway, slow pathway, inferior pyramidal space, infero-septal recess

## Abstract

The anatomical arrangement of the atrioventricular node has been likened to a riddle wrapped up in an enigma. There are several reasons for this alleged mystery, not least the marked variability in structure between different species. Lack of detailed knowledge of the location of the node relative to the atrial and ventricular septal structures has also contributed to previous misunderstandings. Recent studies comparing the findings of gross dissection with virtual dissection of living datasets, combined with access to a large number of serially sectioned human and animal hearts, have served to provide the evidence to solve the riddle. We summarise these findings in this review. We explain how the node is located within the atrial walls of the inferior pyramidal space. It becomes the non-branching component of the atrioventricular conduction axis as the axis extends through the plane of atrioventricular insulation to enter the infero-septal recess of the left ventricular outflow tract. The node itself is formed by contributions from the tricuspid and mitral vestibules, with extensive additional inputs from the base of the atrial septum. We show how knowledge of development enhances the appreciation of the arrangements and offers an explanation as to why, on occasion, there can be persisting nodoventricular connections. We discuss the findings relative to the circuits producing atrioventricular re-entry tachycardia. We conclude by emphasising the significance of the variation of the anatomical arrangements within different mammalian species.

## 1. Introduction

Sir Winston Churchill, when considering the actions of the Soviet Union prior to the commencement of the Second World War, likened the situation to a riddle wrapped up in an enigma. The anatomy of the atrioventricular node has been addressed in comparable fashion [1]. When considering the problems involved, one of us then pointed out that more data would be required if, like Oedipus, we are to solve the riddle, which in the case of Oedipus related to the Sphinx [2]. Over the past two or three years, since we provided our previous review [1], our knowledge has continued to improve. The advances reflect, in part, our increasing experiences in comparing the findings from gross dissection with the information now available from the virtual dissection of living datasets produced using computed tomography. In similar fashion, the evidence available from examination of serially sectioned histological datasets [1] is now enhanced by access to data from hearts imaged down to a few microns using hierarchical phase-contrast tomography (HiP-CT) [3]. Access to this new information has permitted us to improve our knowledge of the location of the atrioventricular node, in particular with regard to the origin of its component parts from the working atrial myocardium, and its insulation as it becomes the non-branching atrioventricular bundle [4,5,6,7]. The information regarding the specific locations of the atrioventricular node relative to the landmarks of the right atrium becomes the more significant as the question is posed as to whether potentials are now being recorded directly from the nodal cardiomyocytes [8]. In this review, we provide an account of our current understanding of the make-up of the atrioventricular node and its relationship to the atrial and ventricular walls. We revisit its development [4,9], discussing these changes in the context of persisting nodoventricular connections. We address the significance of the morphological findings in understanding the anatomical substrates for atrioventricular nodal re-entry tachycardia [10]. We conclude by emphasising again the major differences to be found between the arrangements in the human heart when compared to the situations in species such as the dog, pig, and ox [11,12]. 

## 2. Materials and Methods

For the purposes of our comparisons, we revisited the numerous datasets at our disposal of serially sectioned human hearts stained using the trichrome technique [1]. We then re-evaluated the arrangement shown in a human heart from a 43-year-old adult sectioned in its short axis, again with the sections stained with trichrome, along with a heart from a neonate of 6 months. In terms of gross anatomy, we revisited the large number of photographs we hold showing the features of the atrial and ventricular chambers and the atrioventricular junctions, in the human heart. We compared the findings from the autopsied specimens with computed tomographic datasets obtained from living patients. We also re-examined the datasets now publicly available from hearts scanned using HiP-CT [3]. So as to extend our knowledge of development, in addition to the information now provided from the material housed in the Human Developmental Biology Resource [13], we were able to gain access to several serially sectioned human fetuses obtained over the period from 9 to 17 weeks of development, the sections being made available from the archive of the University of Leiden in the Netherlands.

## 3. Results and Discussions

### 3.1. The Historical Perspective

**Figure 1:**
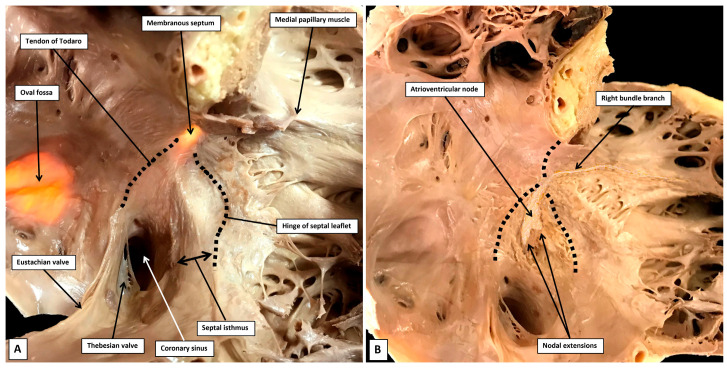
(**A**) Panel A shows the septal surfaces of the right atrium and ventricle of an adult human heart having removed the parietal walls of the chambers. The specimen is transilluminated from the left side to show the locations of the oval fossa and the membranous septum. The dashed lines show the locations of the tendon of Todaro and the hinge of the septal leaflet of the tricuspid valve. As we will describe, these structures form the boundaries of the triangle established by Koch as providing the landmarks to the atrioventricular node (see Figure 2A). The double headed arrow shows the location of the septal isthmus, an area that will become significant when we discuss the substrates for atrioventricular re-entry tachycardia. (**B**) In panel B, careful dissection has revealed the location of the atrioventricular node within the walls of the triangle and shown its continuation as the right bundle branch. When using gross dissection, however, there is no certainty that the pathway demonstrated is the solitary pathway for atrioventricular conduction.

An indication of the problems that relate to atrioventricular nodal anatomy is provided when considering the difficulties that arose around the turn of the 19th century in establishing the very existence of atrioventricular conduction. In this regard, towards the end of the 19th century, it had been established by Gaskell that conduction across the atrioventricular junctions was myogenic rather than neurogenic [14]. The anatomical substrate for atrioventricular conduction, however, remained contentious. Wilhelm His Junior had claimed to have revealed the presence of a solitary pathway related to the membranous component of the ventricular septum [15]. In contrast, Stanley Kent, a physiologist working in London, had claimed to have found multiple muscular pathways crossing the atrioventricular junctions in the normal heart [16]. As we will demonstrate, there is some substance in the findings of Kent, but the structures he identified are not to be found as atrioventricular myocardial pathways in the normal heart. Despite the existence of the evidence provided by His, the anatomist Arthur Keith, working in the first decade of the 20th century, had experienced difficulty in identifying the alleged solitary pathway [17]. Keith, in his initial searches for the conducting bundle, had relied on gross dissection. We now know that it is possible, with our current knowledge, to demonstrate, using the technique of gross dissection, the location of the atrioventricular node and its continuation as the right bundle branch (Figure 1). 

**Figure 2:**
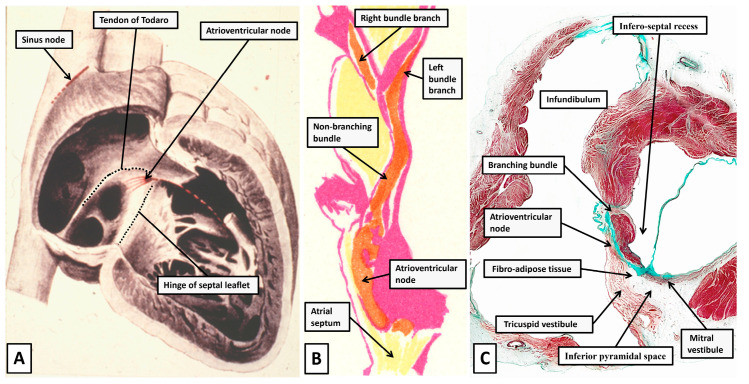
(**A**) Panel A shows the drawing made by Walter Koch [21], in which he shows the boundaries of the triangle we now describe in his name as providing the landmarks to the atrioventricular node (compare with Figure 1). (**B**) Panel B is a reproduction of one of the drawings made by Sunao Tawara to show the atrioventricular node, which he described as the Knoten [19], as the atrial origin of an atrioventricular conduction axis. We have reorientated the figure as it would be seen in the short axis of the ventricular mass, viewed from the apex of the heart looking towards the base. (**C**) Panel C is a section taken in the short axis of an adult human heart. It shows the accuracy of the arrangement as depicted by Tawara. The section also serves to demonstrate the answer to a potential paradox. As viewed in the drawing provided by Koch, the node is seen to be located centrally within the cardiac mass. The short axis shown in panel C confirms that to be the case. At the same time, however, the node is directly related to the epicardial tissues of the atrioventricular grooves. It is positioned within the atrial walls at the apex of the inferior pyramidal space.

When Keith had been dependent on dissections, such as shown in Figure 1, he had not been convinced of the presence of the alleged myocardial pathway joining together the atrial and ventricular myocardial masses [17]. As he described in his autobiography [18], after having made multiple attempts using gross dissection, his scepticism reached such levels that he was constrained to write the letter to the Lancet in which he laid out his concerns [17]. At the time, he was collaborating with James Mackenzie in attempting to resolve the issues of atrioventricular conduction. Mackenzie then shared with Keith the findings described by a Japanese investigator working in the laboratory of Ludwig Aschoff in Marburg, Germany [19]. Keith was not exaggerating when stating that the findings in the monograph, written by Sunao Tawara, ushered in a new epoch of heart research [18]. Keith himself, by then working with Martin Flack, endorsed the findings of Tawara in a study in which these investigators, for the first time, established the location of the sinus node [20]. The achievement of Tawara had been, using serial histological sections from the hearts of several animal species, including human hearts, to show that the bundle described by His was but a part of a much more extensive histologically specialised axis. As was shown by Tawara [19], the axis took its origin from a collection of cardiomyocytes located in the base of the atrial septum. Tawara described the atrial component as a “Knoten”. He showed how the pathway from the Knoten crossed the plane of atrioventricular insulation as the bundle described by His. He further demonstrated how the pathway then branched on the crest of the superior component of the muscular ventricular septum, continuing as insulated fascicles that extended into the ventricular apexes before ramifying with the working ventricular cardiomyocytes. He likened the arrangement to a tree, with its roots in the atrial septum as the “Knoten”, its trunk as the bundle described by His, and the ventricular ramifications as the branches of the tree [19]. Shortly thereafter, another German pathologist, Walter Koch [21], seeking to confirm the findings of Keith and Flack regarding the significance of the sinus node as the origin of the cardiac impulse [20], provided the illustration that has established the significance of the triangle we now describe in his name as providing the gross anatomical landmarks to the Knoten as described by Tawara (Figure 2).

By the end of the first decade of the 20th century, therefore, excellent descriptions had been provided not only of the histological make-up of the atrioventricular node, but also the landmarks that permitted recognition of its location within the heart. Why, then, should the anatomy of the atrioventricular node still be considered to be enigmatic? A possible answer to this specific problem can be perceived in the drawing of Walter Koch [21]. The node is shown as being located centrally within the heart, directly adjacent to the membranous part of the ventricular septum (Figure 2A). Histological sections confirm this to be the case. At the same time, analysis of the histological sections (Figure 2C) shows that not only is the atrioventricular node centrally positioned, but it is also directly related to the fibroadipose epicardial tissues contained within the atrioventricular grooves. This is because the node lies at the apex of the component of the atrioventricular junctions known as the inferior pyramidal space [5,22]. The space had first been brought into prominence by Sealy when surgically correcting the anatomical substrates of the Wolff–Parkinson–White syndrome [23]. The atrial wall of the triangle of Koch forms one of its boundaries. It is an understanding of the anatomy and make-up of the space [5] that further unlocks the mysteries surrounding the enigmatic anatomy of the atrioventricular node. 

### 3.2. The Anatomy of the Inferior Pyramidal Space

**Figure 3:**
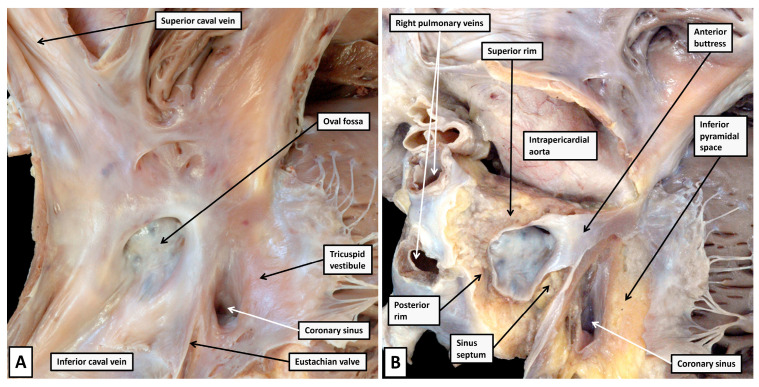
(**A**) In panel A, the potential septal surfaces of the right atrium and ventricle at the level of the atrioventricular junction have been revealed by opening and spreading the junction. (**B**) The dissection shown in panel B has removed all the walls of the right atrium that, on their posterior aspect, are bordered by the fibro-adipose tissues of the extramural areas, themselves enclosed within the epicardial coverings of the heart. The dissection shows how the atrial septum is formed primarily by the floor of the oval fossa, along with its anterior buttress, which binds the floor of the fossa, derived from the primary atrial septum, to the insulating tissues of the atrioventricular junction. The remaining rims of the fossa are infoldings of the atrial walls. The so-called sinus septum, also known as the Eustachian ridge, is similarly an infolding between the walls of the inferior caval vein and the coronary sinus. Removal of the tricuspid vestibule shows how it forms one of the boundaries of the inferior pyramidal space, which is filled with the fibro-adipose tissues of the inferior atrioventricular groove.

The extent of the space, and its relationships, are not immediately evident when the right atrioventricular junction is opened so as to permit analysis of its seemingly septal components (Figure 3A).

**Figure 4:**
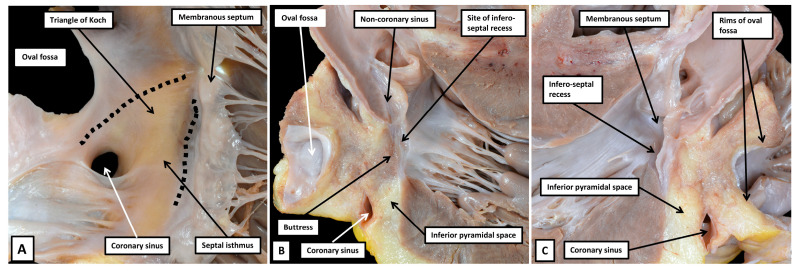
(**A**) The dissection of an infant heart offers further insights to the boundaries of the inferior pyramidal space. To produce the image shown in panel A, a cut had been made to liberate the right atrial wall from its posterior neighbours. (**B**) The cut was directed obliquely into the aortic root, producing the image shown in Panel B, which shows the posterior parts as seen from the right side. The cut has revealed the extent of the fibro-adipose tissues filling the inferior pyramidal space, which is limited cranially by the buttress of the atrial septum. The cut through the aortic root has also revealed the location of the infero-septal recess of the left ventricular outflow tract. Note that the walls of the coronary sinus are surrounded by the fibro-adipose tissues of the inferior pyramidal space. (**C**) Panel C shows the left side of the segment removed by the cut that liberated the right atrial structures from the posterior neighbours. The atrioventricular node is contained within the tissues shown behind the wall of the triangle of Koch as shown in panel A. As we will describe, it penetrates at the level of the membranous septum to enter the infero-septal recess as the non-branching atrioventricular bundle.

As is shown when a dissection is made, removing all those parts of the walls that abut on extramural fibro-adipose tissues (Figure 3B), the atrial septum, in its greater part, is represented by the floor of the oval fossa. This component is the embryonic primary atrial septum. The superior rim of the fossa is still frequently described in many textbooks as representing the septum secundum [24]. Already by the end of the 19th century, however, Röse had established that the rim was no more than an infolding between the walls of the right and left atrial chambers [25]. There is, nonetheless, a second atrial septal component that is formed during cardiac development. As we will show in our section devoted to development, it is produced by muscularisation of the mesenchymal components derived by the ingrowth of tissue through the dorsal mesocardium, known as the vestibular spine, along with the part carried as a cap on the leading edge of the primary septum itself [26]. As we will also show, this structure, which becomes the septal buttress, is of great significance in terms of providing the septal inputs to the atrioventricular node. When assessed in attitudinal fashion, it is located anterior relative to the fossa. When shown in four-chamber sections, however, it is usually depicted as being inferiorly positioned. With this feature in mind, we have tended to describe it as the antero-inferior buttress. It forms the apex of the inferior pyramidal space, which in three dimensions is pyramidal in its extent. The base is formed by the epicardial covering of the diaphragmatic aspect of the heart known as the crux. It is within the crux that the coronary sinus enters the cavity of the right atrium. Its orifice, subsequent to removal of the right atrial wall, can be seen to be surrounded by the fibro-adipose tissues of the pyramidal space (Figure 4).

**Figure 5:**
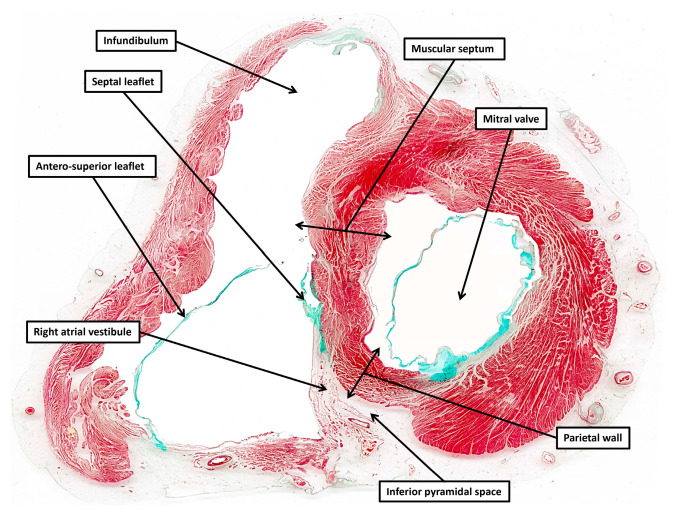
The section is from the same series as shown as panel C of Figure 2. This section shows how the right atrial vestibule is separated by the fibro-adipose tissues of the inferior pyramidal space from the parietal wall of the left ventricle, which forms the ventricular boundary of the space. Compare with Figure 6B.

As is revealed by the dissection shown in Figure 4, the surface of the triangle of Koch, as seen from the right side, forms the right atrial wall of the inferior pyramidal space. The atrioventricular node itself, therefore, housed within the buttress of the atrial septum, can be considered as being located within the pyramid of Koch [5]. The floor of the pyramid is formed by the basal surface of the ventricular cone. This part of the ventricular mass is itself often described as being septal. McAlpine, for example, identified the area as the posterior septal process [27]. The area in question is inferior, rather than posterior. It is also questionable as to whether it is part of the ventricular septum. Serial sections taken through the ventricular cone show that the ventricular walls diverge one from the other as they approach the cardiac crux. Their atrial surface is sloping and is overlaid by the right atrial wall of the triangle. It is possible that parts of the diverging walls do continue to exist as part of the inferior muscular ventricular septum. The histological sections, nonetheless, show that the component opposite the sloping right atrial wall, and forming the ventricular boundary of the inferior pyramidal space, is part of the parietal wall of the left ventricle (Figure 5).

**Figure 6:**
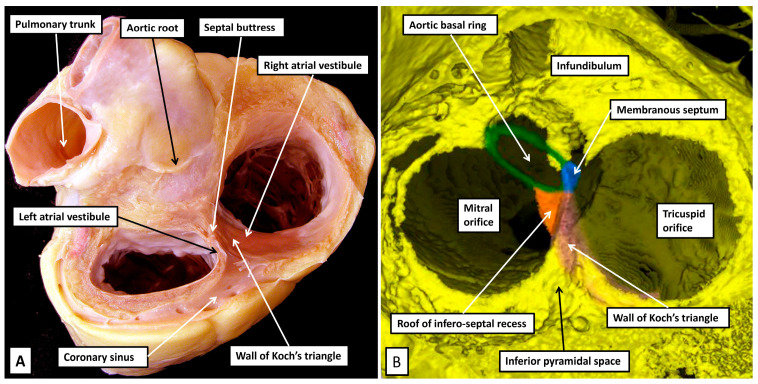
(**A**) Panel A shows the anatomist’s view of the atrioventricular junctions, having cut back the atrial walls to the level of the vestibules. The right atrial wall of Koch’s triangle can be judged to overlie the fibro-adipose tissues of the inferior pyramidal space, with the base of the pyramid formed by the cardiac crux, and its apex by the atrial septal buttress. (**B**) Panel B shows the view that can now be obtained by segmentation of living computed tomographic datasets. The image has been orientated so as to match the view shown in panel A. The wall of Koch’s triangle and the roof of the infero-septal recess have been segmented. As can be seen in panel B, the base of the pyramid is the inferior surface of the heart.

The rightward wall of the space, therefore, is the right atrial surface which, when viewed from the right side, is known as the triangle of Koch. This wall is separated by the fibro-adipose tissues of the space itself from the diverging surfaces of the ventricular cone. The leftward boundary of the space is then formed by the left atrial vestibule, with the base of the pyramid formed by the inferior epicardial surface of the heart itself (Figure 6).

### 3.3. The Make-Up of the Atrial Walls

**Figure 7:**
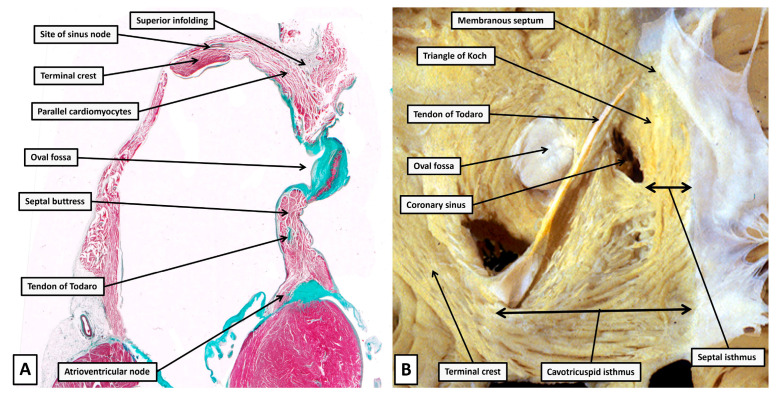
(**A**) Panel A shows a cross section through the walls of the right atrium from a heart obtained from a 6-month-old neonate, with the image orientated in “four chamber” fashion. Even at this low magnification it is possible to recognise the cardiomyocytes making up the atrioventricular node and the site of the sinus node, although the cells of the sinus node are not seen. The remaining walls are made up of aggregated working cardiomyocytes, with an obvious parallel alignment in the right atrial wall making up part of the superior rim of the oval fossa. (**B**) Panel B shows a dissection of the septal surface of the right atrium, having removed the endocardium to show the “grain” produced by the aggregation of the cardiomyocytes. There are obvious parallel alignments in the pathways leading into the triangle of Koch.

As we will show in the next section, it is the right and left atrial vestibules, along with the septal buttress, which come together to provide the area that houses the atrioventricular node. The cardiomyocytes making up the node, however, can be distinguished on the basis of their histological appearances from the cardiomyocytes making up the greater part of the atrial walls. The issue of “specialisation” of the atrial cardiomyocytes has long been contentious. We have already discussed how the location of the node, and its histological make-up, were established by Tawara in the first decade of the 20th century [19]. And we have also discussed how, when endorsing the account provided by Tawara, Keith and Flack described the location, and histological arrangement, of the sinus node [20]. Already by the end of the first decade, a suggestion had been made that a comparable histologically discrete tract could be identified extending between the sinus and atrioventricular nodes. No histological evidence, however, was produced to substantiate the claim [28]. The suggestion was taken sufficiently seriously, nonetheless, to underscore the creation of a session of the German Pathological Society, held in 1910, to discuss the issue. This resulted in the establishment of criterions for the distinction of nodes and tracts [29,30]. Those proposing the criterions suggested that cardiomyocytes, if considered to be forming tracts, should be histologically discrete, traceable from section to section in serially prepared datasets, and be insulated from the adjacent working cardiomyocytes. The cardiomyocytes making up the nodes needed to fulfill only the first two of these criterions. No evidence has been produced to date to question the ongoing validity of these rules when assessing the significance of serially sectioned histological datasets. When assessed on this basis, it can be shown that the atrial walls interposing between the two nodes are made up of working atrial cardiomyocytes, albeit that, in parts of the walls, the working cardiomyocytes are themselves aggregated together so as to favour preferential conduction (Figure 7).

### 3.4. The Histological Make-Up of the Atrioventricular Node

**Figure 8:**
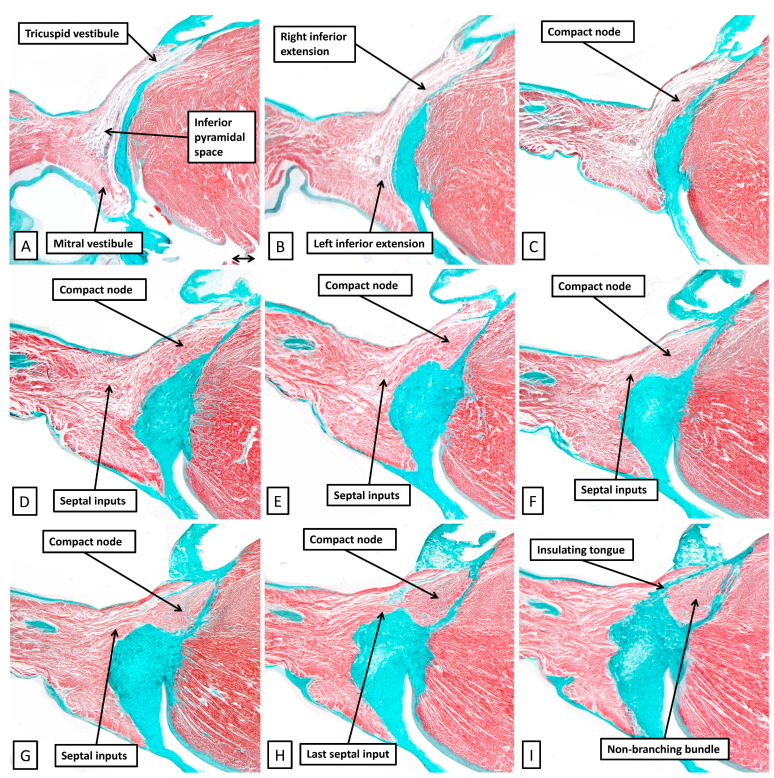
(**A**–**I**) The panels are selected sections from the serially prepared dataset obtained from the heart from the neonate of 6 months shown in lower magnification in Figure 7A. To make this figure, the images are orientated in an attitudinally appropriate fashion, with the right side to the top. Panel A shows a section at the base of the pyramid of Koch, with subsequent sections moving cranially to panel I, which is close to the apex of the pyramid.

By using the serial histological sections from the heart of the 6-month-old neonate used to prepare Figure 7, it is possible to show how the working atrial cardiomyocytes merge to become histologically specialised as they come together to form the atrioventricular node (Figure 8).

**Figure 9:**
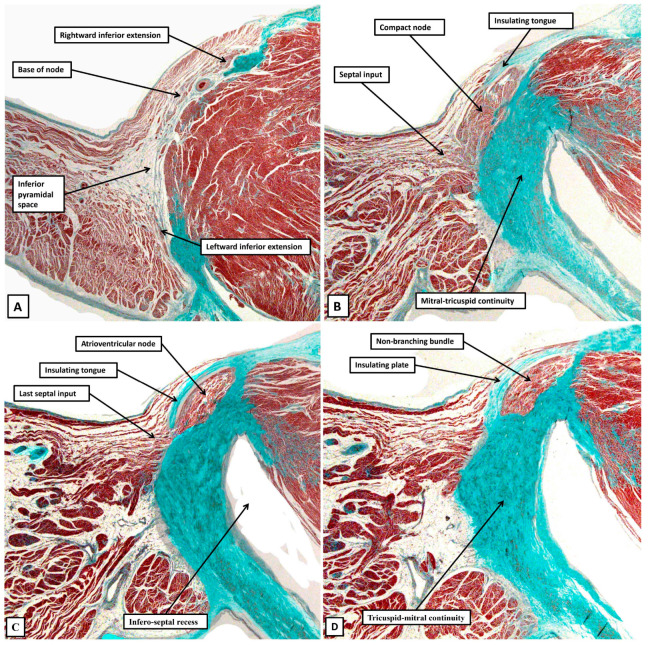
(**A**–**D**) The histological sections, again shown in a serial fashion extending from the base of the pyramid of Koch (panel A) to its apex (Panel D), are taken from an adult heart. The sections are again orientated in an attitudinally appropriate fashion, with the right-sided chambers to the top of the image.

The cardiomyocytes within the tricuspid and mitral vestibules forming the diverging inferior walls of the pyramid of Koch change their character subtly. They can then be recognised as the inferior nodal extensions (panels A and B). The nodal extensions themselves are overlain by the working cardiomyocytes of the vestibules. The rightward extension, occupying the tricuspid vestibule, is longer than that formed within the mitral vestibule. The two inferior extensions themselves then merge to produce the body of the compact node, which is plastered against the fibrous atrioventricular insulating plane (panel C). As the sections are traced cranially, it becomes possible to recognise the cardiomyocytes of the inferior part of the septal buttress merging with the compact component of the node (panel D). The septal inputs are extensive (panels E through G) but gradually taper off as the plane of atrioventricular insulation itself develops as a tongue of fibrous tissue that walls off the compact node from the buttress (Panel H). Once the fibrous tongue has interposed between the atrial cardiomyocytes and the cells of the conduction axis, the axis itself becomes the non-branching part of the ventricular component. When assessed in terms of gross histology, there is no obvious difference in the cardiomyocytes making up the different parts of the node. Nor is there any noticeable difference as the axis transitions from the compact node to become the non-branching bundle. The cardiomyocytes do then become better aligned, and more tightly packed, as the axis continues as the non-branching bundle. And, as we will discuss in our section devoted to atrioventricular nodal re-entry, differences have been described in the connexins forming the gap junctions within the conduction axis. The histological pattern, nonetheless, does not change markedly in terms of the overall arrangement when adult datasets (Figure 9) are compared with the neonatal dataset shown in Figure 8.

In some datasets, as for example the set shown in Figure 8, it is possible to recognise cardiomyocytes at the sites of merging with the working areas that are intermediate in their staining characteristics. These can be described as transitional cells. They are more readily recognised in hearts obtained from younger individuals, although they are not uniformly present [1]. In older individuals, it is frequent to find dispersion of the walls due to infiltration of fibro-adipose tissues, or evidence of ischemic disease. There is also variation in the extent of the septal inputs [1]. These individual changes are found within the context of the same basic build-up, with the two inferior extensions coming together to form the compact node, and with septal inputs adding to the compact component. 

Although there is no obvious difference in the make-up of the node as judged using standard histology, it has been possible to show different parts depending on the gap-junctions between the cardiomyocytes. These changes are potentially significant when considering the substrates for atrioventricular nodal re-entry. Thus, Hucker and colleagues, by using molecular markers for Connexin43, were able to show continuity from the non-branching bundle into the rightward inferior extension [31]. They commented that the findings observed using the molecular markers could not be matched when assessing their sections prepared using standard histology. Their findings can be explained on the basis of our knowledge of development, since the components identified by the presence of gap junctions containing Connexin43 are all derived from the ring of primary cardiomyocytes that surrounds the embryonic interventricular communication [32]. It is pertinent at this stage, therefore, to provide an account of the development of the overall conduction axis.

### 3.5. The Development of the Atrioventricular Conduction Axis 

**Figure 10:**
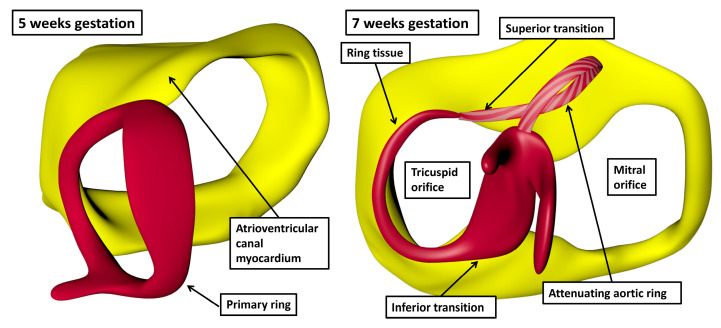
The panels show the key stages in remodelling of the embryonic interventricular communication relative to the formation of the atrioventricular node. The panel seen to the left hand shows a view, as seen from the apex looking towards the base, of the ring of specialised cardiomyocytes that surround the embryonic interventricular foramen prior to expansion of the atrioventricular canal. The panel seen to the right hand shows the arrangement subsequent to expansion to form the right atrioventricular junction, and as the aortic root begins to translocate to achieve its eventual position within the left ventricle. The reconstructions are made from the interactive pdf files available from the publication of Hikspoors and colleagues [33].

Already at the stage at which the ventricular apical components begin to balloon from the ventricular component of the primary heart-tube, and whilst the atrioventricular canal connects only to the developing left ventricle, it is possible to recognise a ring of specialised cardiomyocytes encircling the interventricular foramen (Figure 10, left hand panel) [33,34].

**Figure 11:**
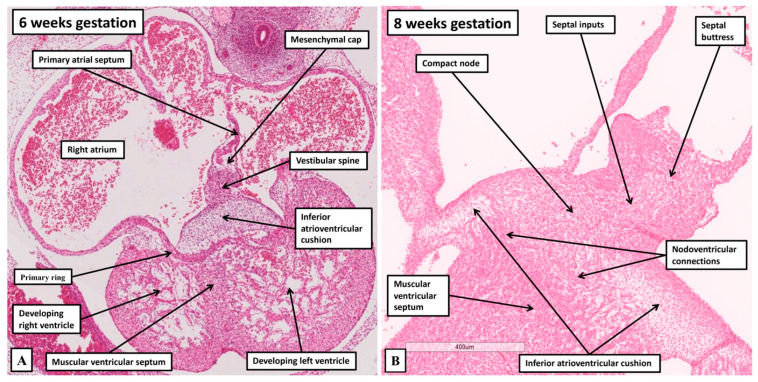
The images show histological sections stained with hematoxylin and eosin, in four-chamber orientation, revealing the arrangement of the primary ring at its inferior transition from the ventricular to the atrial components of the developing heart. (**A**) Panel A shows the arrangement at Carnegie stage 17, equal to around 6 weeks of gestation. At this stage, the vestibular spine and mesenchymal cap have still to muscularise. (**B**) Panel B shows the situation at the end of the embryonic period of development. The spine and cap have muscularised and provide the septal inputs to the compact node, which is derived from the primary ring. The ring extends between the horns of the inferior atrioventricular cushion as the ring itself transitions to become the compact atrioventricular node. At this stage, the insulation between the base of the node and the crest of the muscular septum has yet to be formed.

With expansion of the atrioventricular canal to provide the right ventricle with its own inlet, the dorsal part of the ring encircles the newly formed right atrioventricular junction (Figure 10, right hand panel). With ongoing development, the specialised cardiomyocytes of the ring become sequestrated within the tricuspid vestibule. The component on the crest of the ventricular septum becomes the spine of the atrioventricular conduction axis. When traced dorsally, the ring emerges from beneath the inferior atrioventricular cushion as it transitions from a ventricular to an atrial entity (Figure 11). 

As shown in panel A of Figure 11, the ring is supported on the crest of the muscular ventricular septum at the site of atrioventricular transition. At this relatively early stage, although the primary atrial foramen has closed, the mesenchymal components have yet to muscularise. By the end of the embryonic period, as shown in panel B of Figure 11, the mesenchymal tissues have become the buttress of the atrial septum. At the same time, they provide the septal inputs to the compact atrioventricular node, which has been formed from the part of the primary ring that is now within the atrial component of the heart, emerging from the ventricular component between the diverging dorsal horns of the inferior atrioventricular cushion. It is the inferior atrioventricular cushion that then insulates the part of the ring that will persist as the non-branching atrioventricular bundle from the myocardium of the atrial septal buttress. The base of the compact node, however, as it transitions from the ventricular to the atrial components, has yet to be insulated from the underlying support provided by the muscular ventricular septum. Hence, at this stage, there are multiple myocardial connections persisting between the base of the node and the ventricular septum. The primary ring, therefore, provides the continuity between the non-branching bundle and the base of the atrioventricular node. It continues as the rightward inferior extension. These are the components of the conduction axis that were shown by Hucker and colleagues to form a discrete pathway on the basis of their molecular identity. The gap junctions they identified were made up of Connexin43. This is also the connexin that provides the gap junctions for the working ventricular cardiomyocytes. This creates a potential problem with identifying the rightward inferior extension as part of the slow pathway into the node. Over and above the presence of the primary ring in the rightward inferior extension, however, this part of the node is also derived from the atrioventricular canal myocardium, as is the connection between the compact node and the mitral vestibule. And the atrioventricular canal myocardium is known to be slowly conducting. It has been shown to be capable of generating an adult-type electrocardiogram, with atrioventricular delay, even prior to formation of the definitive atrioventricular node. It is the canal myocardium, therefore, that is likely to provide the major components of the slow pathways, contributing to both the right and left inferior extensions [35]. 

**Figure 12:**
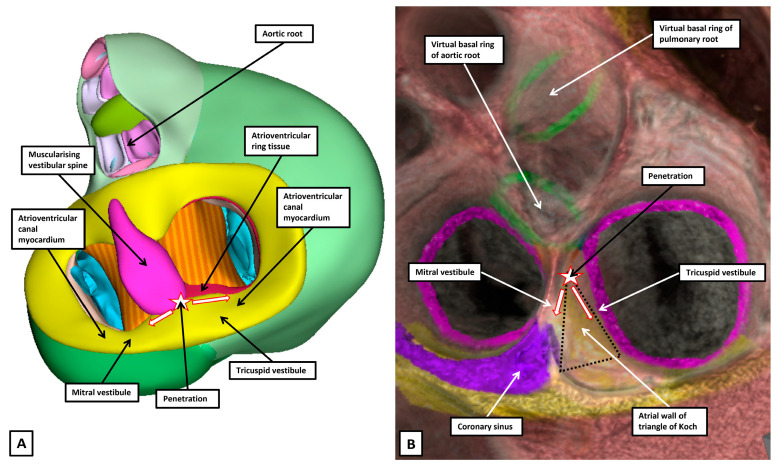
The panels show the major changes required to transform the arrangement at the end of the embryonic period of development (panel A) to the definitive arrangement (panel B). (**A**) Panel A is a reconstruction of an embryonic heart at 8 weeks of development. There has been no expansion of the atrioventricular junctions to produce the inferior pyramidal space, and the aortic root has yet to be “wedged” between the superior parts of the right and left atrioventricular junctions. Note how the primary ring emerges from beneath the inferior atrioventricular cushion to enter the tricuspid vestibule (See also Figure 11B). Both vestibules, however, are derived from atrioventricular canal myocardium, which is slowly conducting. (**B**) Panel B shows a virtual dissection of a computed tomographic dataset from a living patient. It is the expansion of the junctions that produces the triangle of Koch and the inferior pyramidal space. Note how the virtual basal ring of the aortic root has become wedged between the superior components of the junctions.

Note should also be taken of the fact that, at the end of the embryonic period, there has been minimal expansion of the atrioventricular junctions, with minimal formation of the inferior pyramidal space. At this stage, furthermore, the aortic root has yet to be translocated to the cavity of the left ventricle, with limited formation of its infero-septal recess (Figure 12A). It is in the fetal stages of development that the changes take place to produce the definitive arrangement (Figure 12B). As yet, information of these changes is scanty, but it is also during these stages that the atrioventricular insulation develops so as to disrupt the multiple nodoventricular pathways still present at the start of fetal development. (Figure 11B).

### 3.6. Putting the Atrioventricular Node Back into the Heart 

**Figure 13:**
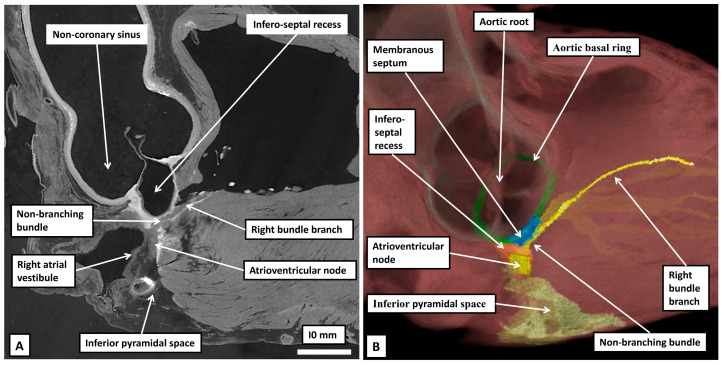
The images are taken from a human heart scanned using HiP-CT technology at the European Synchrotron Radiation Facility, made available at 19.89 um, in this case, of the heart of the body donor s-20-29 [3]. (**A**) Panel A is a two-dimensional image, showing the transition from the atrioventricular node at the apex of the inferior pyramidal space, through the non-branching bundle located within the infero-septal recess, and to the continuation as the right bundle branch. (**B**) Panel B then shows how it is possible to reconstruct these features within the heart itself, showing the relationship of the ventricular components of the axis to the virtual basal ring of the aortic root.

We now know that the atrioventricular node has a uniform make-up, with the inputs from the atrial vestibules reinforced by the contributions made from the atrial septum [1]. And we have now established that it has long been known that the node is located towards the apex of the triangle of Koch [21]. The precise location of the node within the triangle, however, is variable [36]. And the extent of the nodal extensions within the atrial vestibules are similarly known to be variable [31]. Precise knowledge of these relationships would be helpful, particularly for those seeking to ablate the pathways into the node or, alternatively, to avoid the node during their ablative procedures [37]. Accurate recording of nodal potentials would obviously greatly enhance the ability to accurately identify these features [8]. Additional information regarding the relationships can now be provided, at least in autopsied hearts, by means of HiP-CT [3]. This new technique now provides the ability to explore the location of the atrioventricular conduction axis in both two and three dimensions (Figure 13).

**Figure 14:**
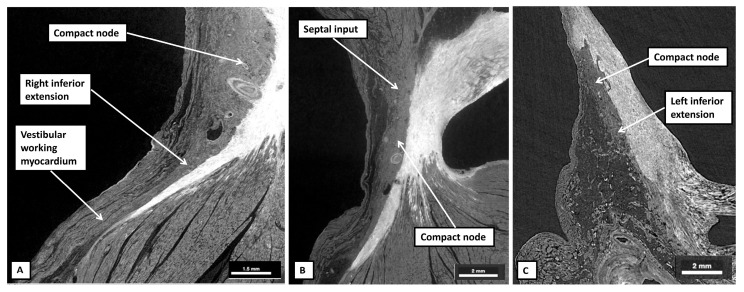
The images are taken from a human heart scanned using HiP-CT at the European Synchrotron Radiation Facility and made available at 6.51 um from the heart of the body donor S20-29. (**A**) Panel A shows the transition between the compact node and the rightward extension into the right atrial vestibule, together with the merge-point between the rightward extension and working myocardium. (**B**) Panel B shows the input to the compact node from the atrial septum, in other words the fast-pathway, with minor infiltration by fat separating it from the right atrial vestibule. (**C**) Panel C shows the much smaller leftward extension from the compact node and the merge-point with the working myocardium of the left atrial vestibule.

Using the same technology, it is now feasible to assess with accuracy the location of the node within the landmarks of the triangle of Koch. The technique also has the potential to show individual cellular morphology. This means that is possible to track the specialised cardiomyocytes of the atrioventricular node as they merge with the working cardiomyocytes of the atrial inputs to the node (Figure 14). At present, only a limited number of hearts are publicly available for exploration, but, as more hearts become available, it will be possible to chart the variability to be found in the make-up of the node and its position within the pyramid of Koch.

### 3.7. The Fate of the Nodoventricular Connections 

**Figure 15:**
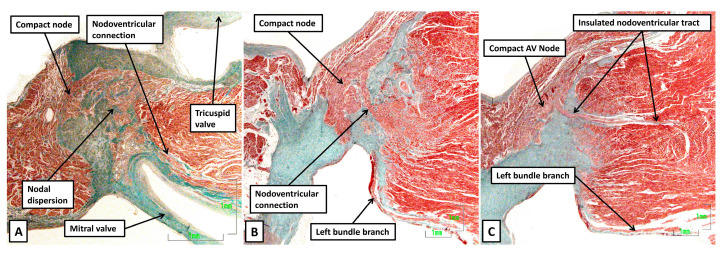
The images show histological sections prepared from three neonatal hearts from patients with Ebstein’s malformation [47]. All sections are orientated in comparable fashion to Figure 7 and Figure 8. (**A**) Panel A shows dispersion of the nodal cardiomyocytes throughout the fibrous atrioventricular insulating tissues, with a strand producing a nodoventricular communication. (**B**) In panel B, there is less dispersion but a more obvious nodoventricular pathway. (**C**) In panel C, there is still less dispersion, but the nodoventricular pathway is insulated as it extends into the crest of the muscular ventricular septum.

We have already shown that, at the end of the embryonic period of development, there are multiple connections between the base of the compact node and the crest of the inferior component of the muscular ventricular septum (Figure 11B). Similar connections between the non-branching bundle and the crest of the muscular ventricular septum are now known to be present almost as uniform findings in the postnatal heart [38]. These pathways connecting with the crest of the muscular ventricular septum were emphasised by Mahaim as providing a “paraspecific” system for atrioventricular conduction [39]. Despite their recognition by Hecht and colleagues as the “superior septal pathways” [40], their potential clinical significance has largely been ignored during the era of the development of cardiac electrophysiology. They have been identified as a potential substrate for ventricular pre-excitation [41]. Although being ubiquitous in humans, however, only occasionally do the pathways lead to ventricular pre-excitation during sinus rhythm [42]. Recent studies, nonetheless, suggested that direct pacing of the atrioventricular conduction axis nearly always results in activation of these dormant connections [43]. Their activation during septal pacing might explain the known electrocardiographic correction of the bundle branch block pattern in patients who undergo pacing in the presumed area of the left bundle branch [44]. The nodoventricular pathways have also been identified as a potential substrate for ventricular pre-excitation [45,46]. But whereas the fasciculo-ventricular pathways are now recognised as potentially being ubiquitous in the postnatal heart, it is rare to find nodoventricular connections persisting postnatally. Such pathways, nonetheless, were found in all six neonatal hearts with Ebstein’s malformation studied histologically (Figure 15) [47]. 

Even in the majority of normal neonatal hearts, in other words without Ebstein’s malformation, it is possible to find some degree of dispersion of the nodal cardiomyocytes within the fibrous insulating tissues of the atrioventricular groove. But it is unusual to find discrete pathways as found in the hearts with Ebstein’s malformation. Such persisting nodoventricular pathways, nonetheless, have been found in patients known to have had ventricular pre-excitation during life [45]. The pathways identified histologically have extended from the base of the compact node, as seen in the patients with Ebstein’s malformation. It is also possible that comparable pathways could be found extending from the rightward inferior nodal extension to the crest of the muscular septum, and hence relatively inferior when considered in the context of their location within the triangle of Koch.

### 3.8. The Substrates for Atrioventricular Nodal Re-Entry Tachycardia

**Figure 16:**
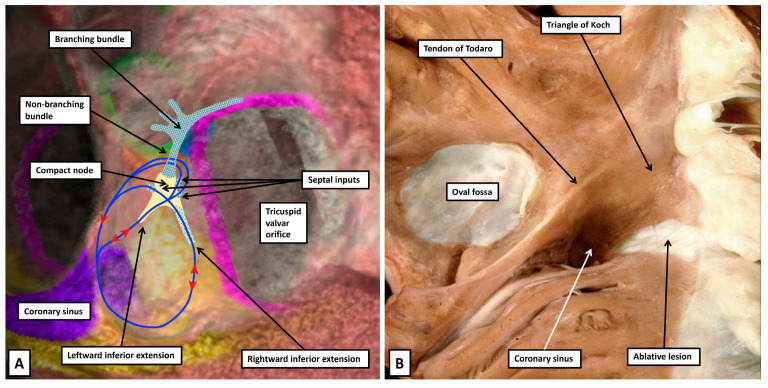
(**A**) Panel A shows the potential circuits for atrioventricular nodal re-entry superimposed on a reconstruction from a living computed tomographic dataset. (**B**) The ideal site for placing an ablative lesion is shown in panel B, where the lesion placed at the septal isthmus terminated the re-entry circuit and cured the tachycardia. Histological examination revealed that the ablation had involved working atrial cardiomyocytes [50].

It has long been known that there are dual pathways composed of working atrial cardiomyocytes that lead into the atrioventricular node [48]. Now described as the fast and slow pathways, it is now also accepted that these connections between the working atrial cardiomyocytes and the specialised cardiomyocytes of the atrioventricular node serve as the inputs and exits for the circuits that underscore atrioventricular nodal re-entry tachycardia. It was initially thought that the re-entry circuit might be contained within the specialised tissues of the node itself. It has become clear that the working cardiomyocytes making up the walls of the pyramid of Koch are also involved in the circuits [12,49]. The initial concept of a substrate involving a lower common pathway was supplanted by categorisation into “slow-slow” and “slow-fast” variants. The approach was then simplified to the concept of typical and atypical tachycardias. All the variants involve the inferior extensions of the atrioventricular node as the terminations of the slow pathway into the node, with the multiple septal inputs functioning as the fast pathway. This approach provides the basis for understanding the various potential circuits (Figure 16A) [50]. It also points to the need to target ablation in most instances on the septal isthmus, albeit that the ablative lesion typically involves working atrial cardiomyocytes. (Figure 16B) [51]. In instances when ablation is ineffective when performed in the septal isthmus, ablation of the mitral vestibule usually produces success. 

### 3.9. The Species Variability in the Morphology of the Atrioventricular Node

**Figure 17:**
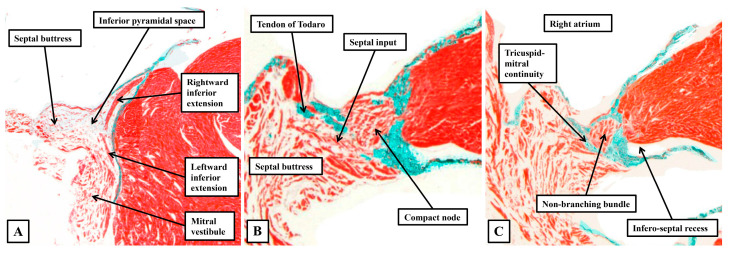
(**A**–**C**) The serial histological sections, prepared in a fashion comparable to the arrangements shown in Figure 7, Figure 8 and Figure 15, reveal that the arrangement of the conduction axis in the murine heart, unlike the situation in the canine and rabbit hearts, is comparable to the arrangement as found in the human heart.

It is often presumed by those conducting animal experiments that the morphology in their chosen species is comparable to the arrangement found in the human heart. This is far from the case. In the rabbit, for example, where the original experiments were performed demonstrating the existence of AN, N, and NH cells [52], it was initially believed that the different cells were all part of the compact atrioventricular node [53]. It subsequently became clear that, following the precedent set by Tawara [19], the area of the conduction axis containing the multiple cell types was the non-branching atrioventricular bundle [54]. It has still to be established why there should be such morphological heterogeneity within the ventricular components of the atrioventricular conduction axis. Equal problems exist, however, when considering the arrangement of the conduction axis in the canine heart. Thus, Racker suggested that, in this species, it was possible to identify proximal and distal atrioventricular bundles [55]. Tawara had illustrated in his initial monograph the fundamental difference between the course of the atrioventricular conduction axis in the canine heart when compared to the human situation [19]. The dog heart, unlike the human arrangement, lacks an infero-septal recess. It is not possible in the canine, therefore, for the conduction axis to penetrate the insulating plane so as to enter directly into the aortic root. In contrast to the arrangement in man, therefore, there is a long non-branching bundle that passes alongside the aortic root within the right-sided tissues. The canine heart also lacks an extensive inferior pyramidal space as found in man, meaning that the atrioventricular node itself has a markedly different architectural arrangement [11]. Thus, whilst the function of the node in producing atrioventricular delay is comparable to the human arrangement, the anatomical arrangement is markedly different. This is not the case for the murine heart. Other species such as the pig, sheep, and ox, as with the dog, lack an infero-septal recess [12]. In these species also, therefore, the non-branching bundle is long and skirts the aortic root on its right side. The mouse, in contrast, does possess an infero-septal recess. And, even though the membranous septum is far less well formed in the murine heart than in man, the conduction axis is still able to pass from the apex of the triangle of Koch to enter the subaortic outflow tract. As in the human heart, the node itself is made up of inputs from both atrial vestibules and the buttress of the atrial septum (Figure 17).

The arrangement as found in the murine heart is then the more interesting, since as in the human heart, there are ubiquitous superior septal pathways joining the non-branching bundle with the crest of the muscular ventricular septum [56]. In the murine heart, furthermore, it is known that ventricular conduction takes place from base to apex, unlike the apex-to-basal pattern observed in the human heart [57]. All of this information makes the murine heart an excellent test-bed for ongoing studies to correlate the morphology and electrophysiology of atrioventricular conduction. 

## 4. Conclusions

We have provided an extensive review of the anatomy of the atrioventricular node. With the advent of virtual dissection of living datasets prepared using computed tomography, and the ability to reconstruct the node with total accuracy using HiP-CT, the architectural arrangement should no longer be considered enigmatic. The findings do no more than attest to the accuracy of the initial description provided by Tawara in 1906 [19]. They also serve further to highlight the accuracy of the work of Koch, who endorsed Tawara’s findings at the end of the first decade of the 20th century [21]. It is arguable that the alleged mystery surrounding the histological make-up of the node could have been avoided had we simply followed their accounts. This is the more so, since excellent criterions had been provided by the end of the first decade of the 20th century to distinguish between working and histologically specialised atrial cardiomyocytes [29,30]. No evidence has subsequently been provided to doubt the ongoing validity of these criterions for those depending on routine histological sections to reveal the arrangement of the cardiomyocytes. Combined with knowledge of development, the findings now provide a suitable background to appreciate the anatomical substrates for atrioventricular nodal re-entry. They also reveal the role of persisting nodoventricular pathways in providing a potential substrate for ventricular pre-excitation. Unlike the fasciculo-ventricular pathways, which produce ubiquitous superior septal pathways, the connections from the compact node to the base of the muscular ventricular septum are not usually found in normally structured hearts. 

## Data Availability

The histological datasets of the human hearts were prepared by Professor Sánchez-Quintana and Nevado-Medina, and they are able to make PowerPoints available to interested parties by direct communication. The developmental material can be accessed via the website of the Human Developmental Biology Resource.
